# Aging-related changes in the diversity of women’s skin microbiomes associated with oral bacteria

**DOI:** 10.1038/s41598-017-10834-9

**Published:** 2017-09-05

**Authors:** Nakako Shibagaki, Wataru Suda, Cecile Clavaud, Philippe Bastien, Lena Takayasu, Erica Iioka, Rina Kurokawa, Naoko Yamashita, Yasue Hattori, Chie Shindo, Lionel Breton, Masahira Hattori

**Affiliations:** 1 Nihon L’Oreal Research & Innovation, KSP, Sakado, Takatsu, Kawasaki, Kanagawa 213-0012 Japan; 2 Laboratory of Metagenomics, Graduate School of Frontier Sciences, The University of Tokyo, 5-1-5 Kashiwanoha, Kashiwa, Chiba 277-8561 Japan; 3 Department of Microbiology and Immunology, Keio University School of Medicine, 35 Shinanomachi, Shinjuku-ku, Tokyo 160-8582 Japan; 4 L’Oreal Research and Innovation, Aulnay-sous-Bois, France; 5 Graduate School of Advanced Science and Engineering, Waseda University, 3-4-1 Okubo Shinjuku-ku, Tokyo, 169-8555 Japan

## Abstract

Skin aging is associated with changes in cutaneous physiology including interactions with a skin microbial community. A striking alteration and diversification in the skin microbiome with aging was observed between two different age groups of 37 healthy Japanese women, i.e. younger adults of 21–37 years old and older adults of 60–76 years old, using bacterial 16S rRNA gene sequencing. The analyses revealed that the alpha diversity/species richness was significantly higher in the older than the younger group for the cheek and forehead microbiomes, while the beta diversity in the overall structure significantly differed particularly for the forearm and scalp microbiomes between the two age groups. Taxonomic profiling showed a striking reduction in the relative abundance of the majority skin genus *Propionibacterium* in the cheek, forearm and forehead microbiomes of the older adults, and identified 38 species including many oral bacteria that significantly differentiated the two age groups with a skin site dependency. Furthermore, we found chronological age-related and unrelated skin clinical parameters that correlate with the observed changes in the skin microbiome diversity. Thus, our data suggested that the diversification of skin microbiomes in adult women was largely affected by chronological and physiological skin aging in association with oral bacteria.

## Introduction

Our body’s largest organ, skin, is covered with various microorganisms, including fungi, bacteria, archaea and viruses^1^, among which bacteria seem to outnumber others^2, 3^. Several studies have revealed that healthy skin microbiome is determined by skin micro-biotopes that include sebaceous, moist and dry environments^4, 5^. In addition to these site effects, various host-related factors such as age and gender are also shown to affect skin microbiome of healthy individuals^4^. Skin disorders such as atopic dermatitis^6^, dandruff^7, 8^ and vitiligo^9^ also affect the subjects’ skin microbiome.

Age-related changes in skin structure and function are attributable to combinations of endogenous intrinsic factors, e.g. cellular metabolisms, immune activity, hormone condition and exogenous environmental factors, e.g. exposure to sun, pollutants, other toxins^10, 11^. Skin aging is characterized by a decrease in sweat, sebum and the immune functions thus resulting in significant alterations in skin surface physiology including pH, lipid composition and sebum secretion^12–15^. These physiological changes provide potential alterations in the skin ecology that may affect the skin microbiome^16, 17^. Several reports have been published on skin microbiomes of longitudinal samples from individuals ranging from 10 s to 30 s of age^6^, transition of skin microbiomes in early life from infancy to adolescence^18–20^ and differences according to the origin of the population and living environment in adult skin microbiome^21, 22^. However, few studies have been conducted on the effect of aging on skin microbiome in various skin sites.

In this study, we focused on the skin microbiome of only women to exclude considerable differences in the ecosystem of skin microbiomes between both genders^23^. Skin bacterial diversity and composition were characterized and compared between two different age groups: younger (23–37 y) and older (60–67 y) Japanese women. Samples from four skin sites, representative of dry (volar forearm) and sebaceous (scalp, forehead, cheek) skin areas were analyzed. The bacterial communities were evaluated based on the 16S rRNA gene sequence data obtained by high-throughput pyrosequencing.

## Results

### Sample collection and sequencing of 16S rRNA gene V1-V2 region

A total of 37 healthy women, free from cutaneous disorders such as acne or atopic dermatitis, of two age groups; 18 subjects aged 23–37 y (younger group) and 19 aged 60–76 y (older group), were recruited. Clinical parameters of their skins were collected (Supplementary Table S1). The bacterial communities were analyzed in 148 skin samples from four skin sites (scalp, forehead, cheek and volar forearm) of all subjects (see Methods). The bacterial 16S rRNA gene V1-V2 region (16S) was sequenced using the 454 GS FLX Titanium platform to obtain a total of 1,019,132 reads after quality and size filtering. For the 148 samples analyzed, the total number of reads per subject per skin site from each age group ranged from 2,601 to 25,904 with a mean of 6,886 reads per sample. To avoid biases caused by differences in sequencing depth of samples, analyses were conducted on 2,500 high-quality reads randomly selected for each sample.

Clustering of the high-quality 16S reads with a ≥96% identity threshold generated a total of 4,156 operational taxonomic units (OTUs) from all samples (Supplementary Table S2). Rarefaction curves of the number of non-redundant OTUs (≥0.1% relative abundance) detected with the increase in the number of individuals showed that the number of OTUs detected in ≥2% (individual-specific) of the all subjects were almost plateauing at the 37^th^ individual for scalp, forehead, and cheek, while that for the forearm sample was still increasing even at the 37^th^ individual but showed almost plateauing in ≥5% (at least two individuals) of all subjects (Supplementary Fig. S1). These data indicated that the sample size of the four skin sites was sufficient to enumerate and cover OTUs shared with at least two individuals in the 37 volunteers.

### Differences in alpha diversity in skin microbiomes between the younger and older groups

**Figure 1:**
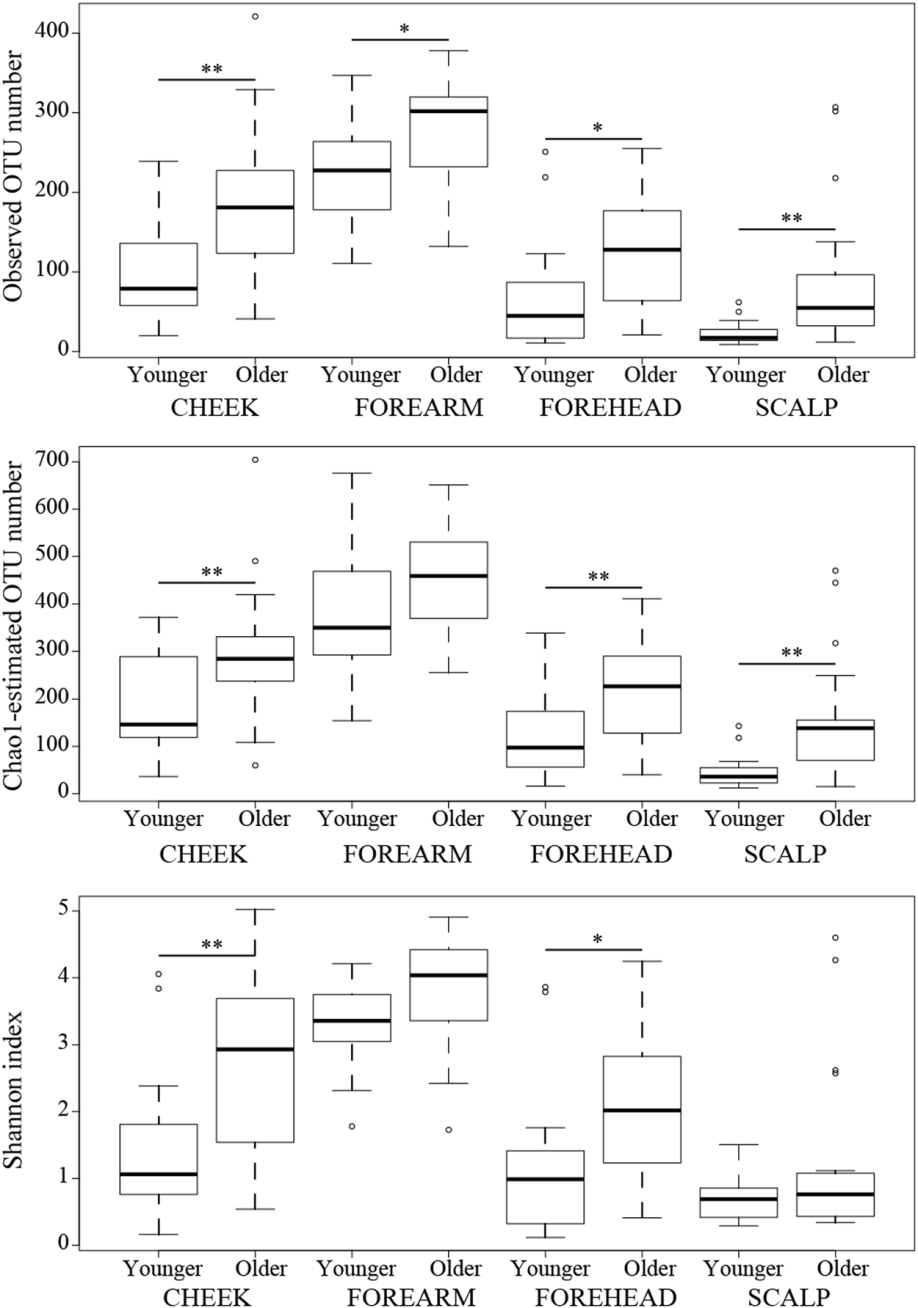
Comparison of alpha diversity of four skin microbiomes between the younger and older groups. Differences in alpha diversity in each skin microbiome between the two age groups (n = 18, younger; n = 19, older group) are shown by three indices. Line in box is a median of index scores, boxes represent interquartile range, whiskers represent lowest and highest values, and dots represent outliers. Statistical significance is indicated by **p < 0.01 and *p < 0.05 (Welch’s *t* test).

We first analyzed alpha diversity (species richness) in the four skin microbiomes based on the observed and Chao1-estimated OTU number, and the Shannon index (Supplementary Fig. S2a). The three metrics analyses gave similar results, revealing that the forearm microbiome had the highest alpha diversity, as suggested by the rarefaction curve, followed by the cheek and forehead, both of which had similar alpha diversity, while scalp microbiome showed the lowest alpha diversity. To further elucidate the age group-specific characteristics of each skin microbiome, we compared the alpha diversity between the younger and the older groups (Fig. 1). The analyses based on the observed OTU numbers showed that the species richness was significantly higher in the older group than the younger group for all the tested sites; forehead (*P* = 0.0169), cheek (*P* = 0.0030), forearm (*P* = 0.0320) and scalp samples (*P* = 0.0059). Similarly, the older group had the higher Chao1-estimated OTUs and the Shannon indices than the younger group for all the skin microbiomes. These data indicated that the older group showed a tendency toward a higher alpha diversity than the younger group for all the skin microbiomes. Particularly, the cheek and forehead microbiomes showed statistical significant differences in the alpha diversity calculated using the all three metrics between the two age groups.

### Differences in beta diversity in skin microbiomes between the age groups

The overall structural similarity and variation (beta diversity between the samples) between the microbiomes from the four skin sites were then examined using the UniFrac-Principal Coordinates Analysis (UniFrac-PCoA) and the UniFrac distance analysis^24^. Both weighted and unweighted UniFrac-PCoA showed that the skin samples tended to be clustered according to skin sites for scalp, cheek/forehead, and forearm (Supplementary Fig. S2b and c). Assessment of these differences by the Permutational Multivariate Analysis of Variance (PERMANOVA) followed by permutation test for homogeneity of multivariate dispersion using the function “adonis” and “betadisper” in R revealed a significant dissimilarity, except for a pair of cheek and forehead microbiomes in neither weighted nor unweighted UniFrac distance (Supplementary Table S3).

**Figure 2:**
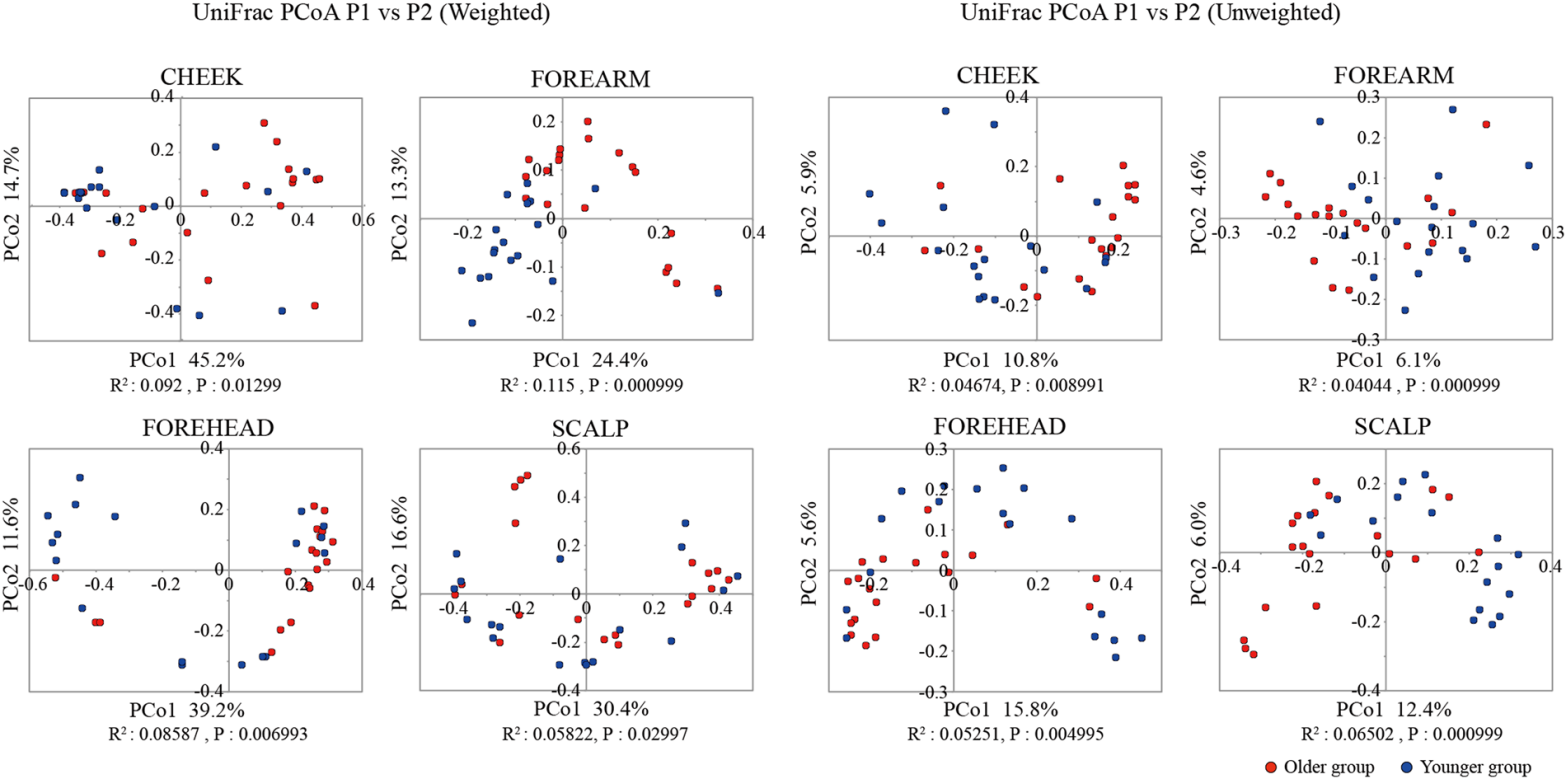
UniFrac-PCoA of 37 skin microbiome samples. The weighted and unweighted UniFrac-PCoA for each skin microbiome is shown, respectively. Red and blue circles represent samples from older and younger subjects, respectively. R-values and p-values of ANOSIM between the two age groups in each skin location are shown below each graph.

The UniFrac analysis to explore differences in the beta diversity between the two age groups for each skin microbiome showed significant differences in all four skin sites (Fig. 2, PERMANOVA P < 0.05). The highest segregated profiles between the younger and older groups were observed in the forearm microbiome in the weighted (PERMANOVA R^2^ = 0.115), and in the scalp microbiome in the unweighted analysis (PERMANOVA R^2^ = 0.06502) (Fig. 2). These data suggested an age-related alteration in beta diversity of the forearm and scalp microbiomes, and that changes in the abundance and membership of community’s species contributed to the age-related diversification of the forearm and scalp microbiomes, respectively. The contribution of the species membership to the difference in the beta diversity of scalp microbiomes was largely due to a remarkable increase in the observed OTU numbers (~3.8 fold of those of the younger group’s samples) in the older group, as compared to those of the other three site microbiomes (0.9~1.6 fold) (Supplementary Table S2). Furthermore, we found that numbers of minor species/OTUs consisting of a small number of 16S reads were characteristically increased in the scalp microbiome of the older group, while almost no apparent increase in the minor species/OTUs was observed in the other three site microbiomes of the older group (Supplementary Fig. S3).

### Differences in taxonomic profiles of skin microbiomes between the age groups

The changes in taxonomic profiles in the age-related diversification of the four skin microbiomes were next investigated. The OTUs were assigned to taxonomies according to the phylotypes in the microbial 16S rRNA gene databases. The phylum-level assignment showed that four major phyla, Firmicutes, Bacteroidetes, Actinobacteria, and Proteobacteria dominated the four skin microbiomes. Among them, Actinobacteria was most predominant, accounting for the average abundance of 61.8%, 40.3%, 67.6%, and 83.3% in the cheek, forearm, forehead, and scalp microbiomes of the all subjects, respectively (Supplementary Fig. S4a). Comparing the abundance of the four phyla between the two age groups revealed that Actinobacteria showed a significantly lower abundance in the cheek, forearm and forehead microbiomes in the older than the younger group with a concurrent increase in the other three phyla (Supplementary Figs S4a and b). The reduction in the relative abundance (~0.6 fold) of Actinobacteria in the older forearm microbiome was most striking. The forearm and scalp microbiomes showed a significantly higher abundance in Proteobacteria in the older than the younger group. The forehead microbiome showed a significantly higher abundance in Firmicutes and Bacteroidetes in the older than the younger group. The cheek microbiome showed a significantly higher abundance in Bacteroidetes in the older than the younger group. Overall, these data indicated global taxonomic differences in the four skin microbiomes between the two age groups.

**Figure 3:**
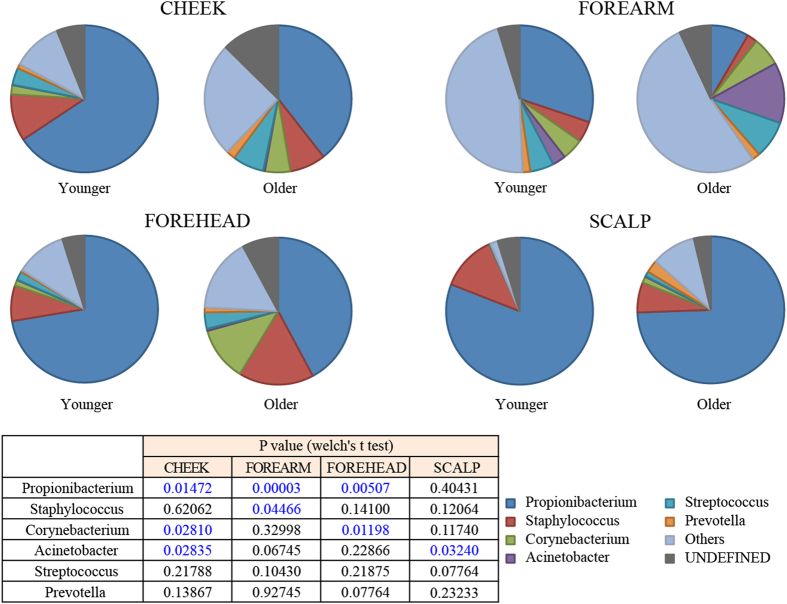
Comparison of the relative abundance of major bacterial genera. The relative abundance of six major genera in the four skin microbiomes is compared between the younger and older groups. Statistically significant p-values are shown in blue.

At the genus level, six genera, *Propionibacterium*, *Staphylococcus*, *Corynebacterium*, *Streptococcus*, *Acinetobacter*, and *Prevotella* were found in larger proportion with the average abundance of ≥1% in the all microbiomes. Among them, the *Propionibacterium* genus comprised the majority of bacterial species, accounting for ~78%, 57%, 52%, and 19% of the total reads in the scalp, forehead, cheek, and forearm samples, respectively (Supplementary Fig. S5). Comparative analysis of the abundance of six dominant genera between the two age groups showed that the abundance of *Propionibacterium* was significantly lowered in the older group as compared to the younger group in the cheek, forearm, and forehead microbiomes (Fig. 3). This was consistent with the marked reduction of Actinobacteria in these three microbiomes of the older subjects since *Propionibacterium* is the majority genus belonging to this phylum (Supplementary Fig. S4). In addition, the abundance of *Staphylococcus* in the forearm was significantly decreased in the older group compared to the younger group, while *Corynebacterium* in the cheek and forehead microbiomes was significantly increased in the older group. *Acinetobacter* was the dominant genus only in the forearm microbiome and significantly increased in the older forearm and scalp microbiomes compared to the younger ones. *Streptococccus* and *Prevotella* also showed higher abundance, albeit not significant, in the older than the younger microbiomes (Fig. 3).

**Figure 4:**
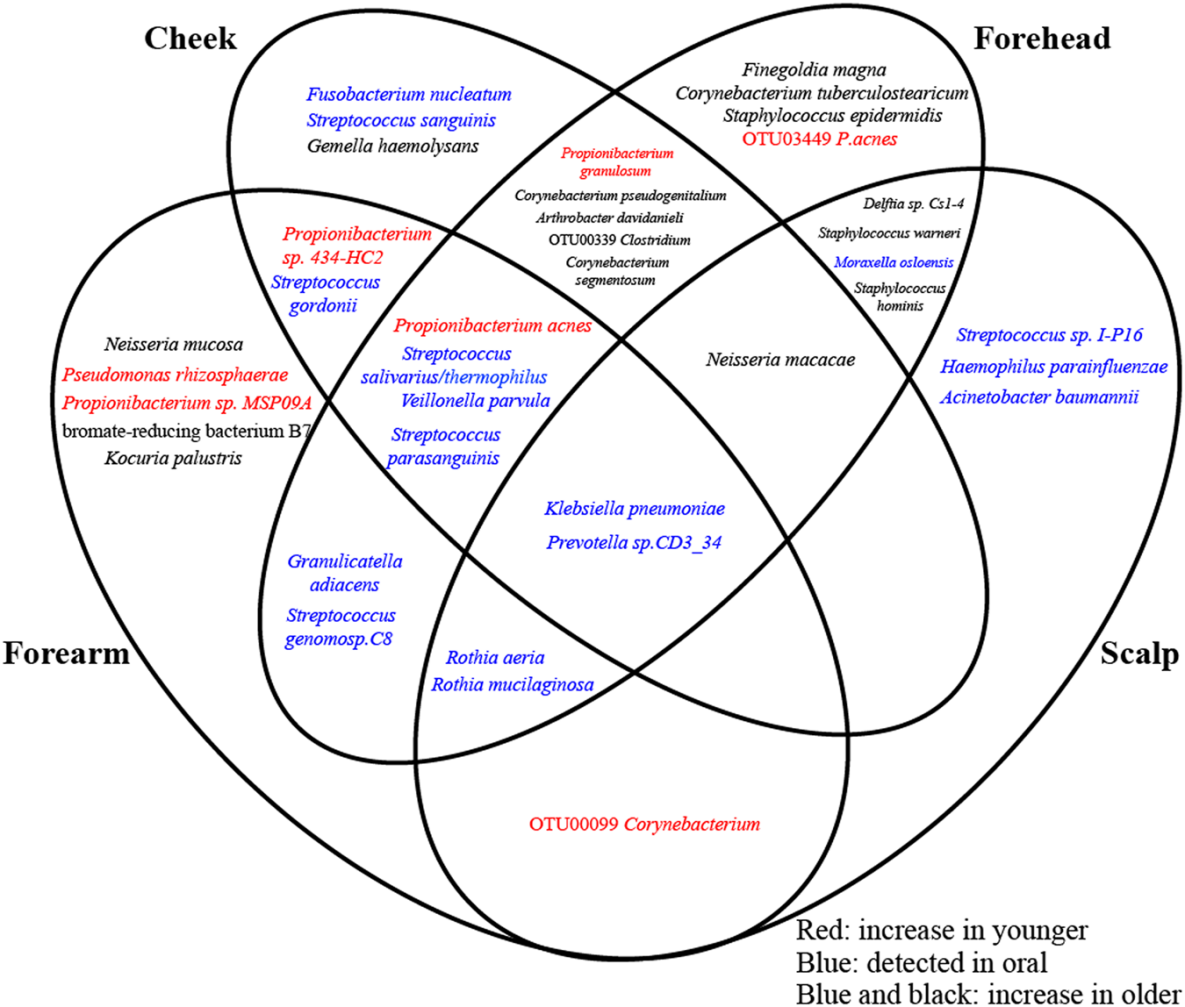
Spatial distribution of the 38 species having significant changes in the abundance between the younger and older groups in the four skin sites. The results shown in Supplementary Table S4 are summarized in the Venn diagram.

**Figure 5:**
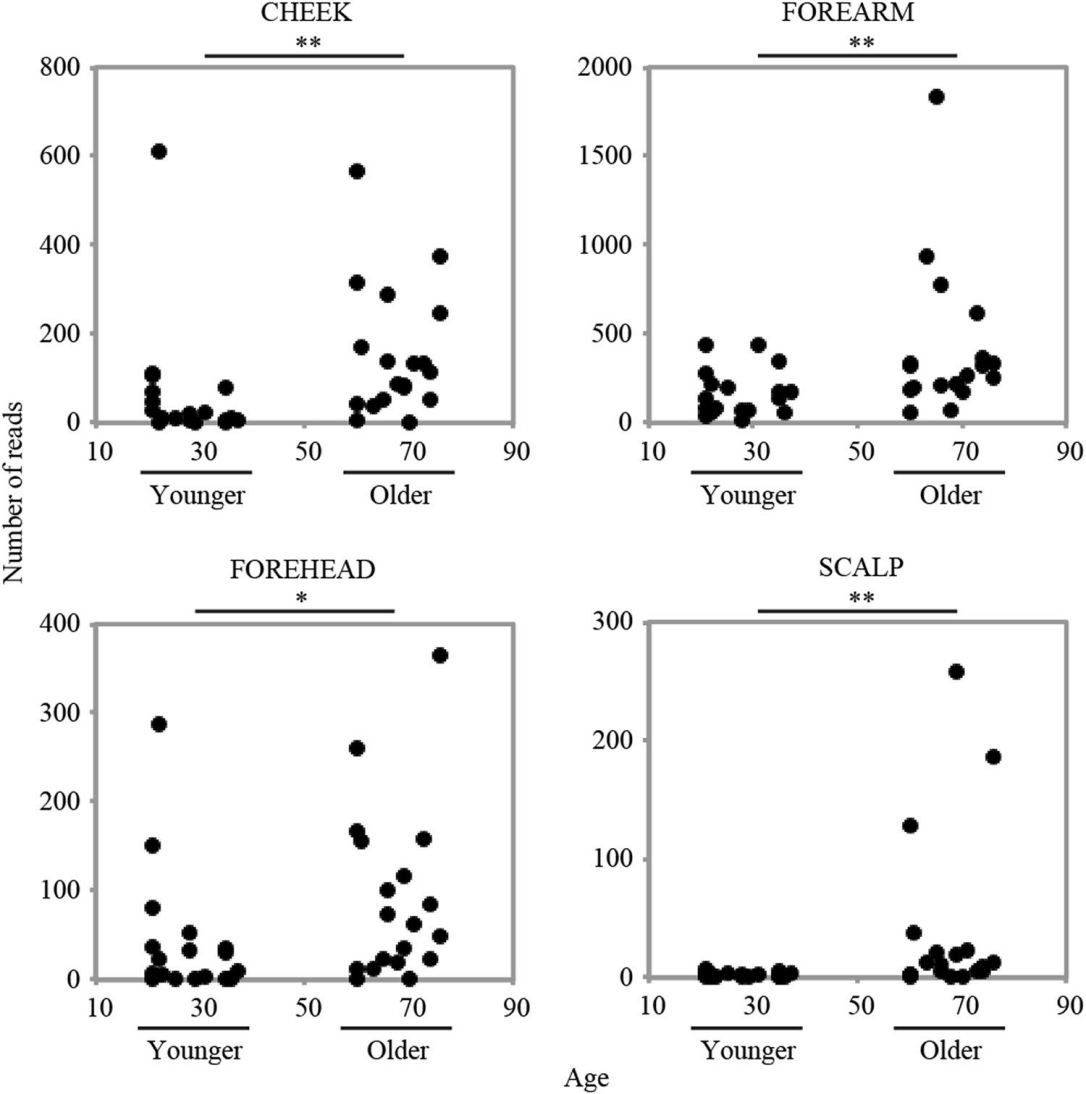
Proportion of oral bacteria in the four skin microbiomes of the younger and older groups. Each dot indicates each subject’s sample. The y-axis indicates the number of reads assigned to the oral bacteria in a total of 2,500 reads per subject while the x-axis indicates subjects’ age. P-values are calculated by Mann-Whitney test, and indicate *p < 0.05, **p < 0.01.

To further identify the species contributing to the age-related difference in the skin microbiomes between the two age groups, we searched for the species/OTUs with ≥0.1% abundance in >50% of the all subjects in at least one skin site by using the linear discriminant analysis (LDA) effect size (LEfSe) method^25^. The analysis identified a total of 38 species/OTUs showing significant changes in the LDA score between them, of which 31 species were overrepresented in the older skin microbiomes, and other seven species, five of which were *Propionibacterium* species, were enriched in the younger skin microbiomes (Supplementary Table S4). Regarding the skin sites, 19, 18, 22, and 13 species were significantly changed in the cheek, forearm, forehead, and scalp microbiomes, respectively. Of them, 15 species showed a skin site-specific change; three in the cheek, five in the forearm, four in the forehead, and three in the scalp microbiome. The other 23 species were altered in more than two different skin sites and only *Klebsiella* and *Prevotella* were enriched in all of the skin sites of the older group (Fig. 4). To determine the skin specificity of these 38 species, the similarity search was performed against the CORE database compiling the species detected in the oral cavity^26^. The results indicated that 16S sequences of 30 species had high similarities of ≥97% with those of the species in the CORE database. Of them, we considered 16 species to be normal oral bacteria, which hit to sequences of ≥3 different CORE IDs (Supplementary Table S4). The 16 species belonging to *Streptococcus*, *Rothia*, *Veillonella*, *Prevotella*, *Fusobacterium*, and *Haemophilus*, all of which were members of the core taxa of the oral bacterial community^27^, were significantly enriched in the older skin microbiomes of the four sites (Fig. 5).

### Relationship between bacterial diversity and clinical skin parameters

To know which skin conditions are associated with the aging-related diversification in the skin microbiome, we conducted a correlation analysis of several bacterial data with chronological ages and clinical skin parameters of the subjects including echogenicity, skin thickness, pore area, and dark spot area in the cheek, and skin physiological parameters such as sebum level and pH of skin surface of the forehead (Supplementary Table S1 and Table 1). Among the cheek parameters, pore density (number of visible pores per unit area) and skin elasticity-related parameters, Ur (immediate retraction of the skin) and Ur/Ue (skin elasticity)^28^ were negatively correlated with the age of subjects, whereas spot density (spot number per unit area) and echogenicity in deep dermis were positively correlated with the age of subjects with statistical significance. In the forehead, sebum level was negatively correlated with the age of subjects as well. Many of these age-associated skin parameters also showed significant correlations with the observed and Chao1-estimated OTU number, the Shannon index, and the relative abundance of several major genera. On the other hand, dark spot area showing no strong correlation with age was positively correlated with the Shannon index and negatively with the abundance of *Propionibacterium* in the cheek with statistical significance (Table 1).

**Table 1:** Correlation analysis between microbial data and clinical skin parameters in the cheek (CK) and forehead (FH)

Site	Parameter	Age	OTU#	Shannon index	Propionibacterium	Staphylococcus	Streptococcus	Corynebacterium	Prevotella
CK	Ur	**−** **0.582**	**−** **0.476**	**−** **0.482**	0.317	−0.127	−**0.373**	−**0.373**	−**0.500**
	Pore Density	−**0.579**	−0.226	−**0.329**	0.244	−0.064	−**0.336**	−0.186	−**0.380**
	Ur/Ue	−**0.482**	−0.245	−0.250	0.224	0.119	−0.178	−0.119	−0.225
	Echogenicity at superficial dermis	−0.171	0.130	0.143	0.031	−0.039	0.198	0.026	0.008
	Ue	−0.133	−0.198	−0.190	0.078	−0.279	−0.204	−0.185	−0.264
	pH	−0.124	0.042	0.014	0.026	0.102	−0.114	0.090	−0.113
	Dark spot area	0.103	0.270	**0.374**	−**0.499**	0.220	0.301	0.182	**0.328**
	Thickness of skin	0.184	−0.187	−0.168	0.042	0.110	−0.149	0.099	−0.167
	Largest pore area	0.227	−0.236	−0.236	0.163	0.011	−0.133	0.086	−0.141
	Pore area average	0.294	−0.274	−0.251	0.211	0.034	−0.125	−0.071	−0.088
	Dark spot density	**0.482**	0.266	0.190	−0.128	−0.042	0.146	0.268	0.162
	Echogenicity at deep dermis	**0.664**	0.485	**0.475**	−**0.378**	0.038	0.284	**0.371**	**0.419**
	Age	**1.000**	0.355	**0.402**	−**0.435**	0.144	0.232	0.417	0.370
FH	Sebum	−0.365	−0.687	**−0.685**	0.440	−0.127	−**0.698**	−**0.492**	−**0.727**
	pH	−0.176	−0.198	−0.204	0.151	−0.059	−0.258	−0.176	−0.150
	Age	**1.000**	**0.339**	**0.397**	−**0.494**	**0.384**	0.224	**0.490**	0.260

Values in the boxes indicate Spearman’s rank correlation coefficients (minus: negative correlation). Correlation coefficients with P < 0.05 are shown in bold.

## Discussion

The present study revealed that the skin microbiome structures of the adult women were strongly affected by skin locations in agreement with the previous studies^4, 5^. A high similarity in the species richness and bacterial composition was found between the cheek and forehead microbiomes. Forearm microbiome showed the highest alpha diversity and scalp the lowest (Supplementary Fig. S2a), which was also similar to the previous reports revealing higher bacterial diversity in dry areas such as forearm than in sebaceous areas such as scalp, forehead, cheek^29–31^. These data can be reasonably explained by a difference in oily and dry nature^32^, and also by the spatial proximity between forehead and cheek on the face.

Our data revealed higher alpha diversity/species richness in older than younger skin (Fig. 1). Similarly, a high skin microbiome diversity was reported in both palms, both forearms and forehead of the older subjects (51–90 y), as compared to the younger subjects (21–50 y) in a cohort of Chinese volunteers^21^. In contrast, another Chinese study showed a greater diversity in skin microbiomes of back of hands, interdigital web space, volar forearm, antecubital fossa, nares, glabella and back in younger (25–35 y) than older subjects (50–60 y)^22^. These discrepancies suggested that the profile of age-related changes in the skin microbiomes depends upon skin locations, but the difference in the lifestyle, living environments, and genders of the study subjects may have to be also considered.

Age-related differences in the beta diversity of the four skin-site microbiomes were more striking for the forearm and scalp than the cheek and forehead microbiomes (Fig. 2). The difference in the scalp microbiome was largely derived from the species membership, which was represented by a marked increase in the number of minor species in the older scalp (Supplementary Table S2 and Fig. S3). On the other hand, the difference in the forearm was largely due to the species abundance, which can be explained by a remarkable reduction of the most predominant genus *Propionibacterium* in the older forearm microbiome (Fig. 3). Thus, these data suggest that the age-related alteration of the skin microbiome is skin site dependent.

Although the difference in the OTU/species numbers between the younger and the older adults was most striking in the scalp among the four skin sites, the difference in the Shannon index was not statistically significant in the scalp (Fig. 1). This may be due to less change in the species evenness of scalp microbiome composed of an extremely high abundance of *Propionibacterium* (~80%). Instead, as described above, the increase in the minor OTU/species numbers may reflect the overall structural change (Supplementary Fig. S3).

In forehead, cheek and forearm, the increase in diversity of the older skin microbiome was concurrent with a significant decrease in the relative abundance of *Propionibacterium* in the older group (Fig. 3). This may be related with a decrease in sebum secretion level in the older skins^33^. The decrease of sebum is normally observed in women due to hormonal changes during and after menopause^14, 34, 35^. In this study, the average sebum secretion level in forehead was found about half lower in the older than the younger group (Supplementary Table S1), and a positive correlation was observed between the sebum level and the *Propionibacterium* abundance in forehead (Table 1). In agreement with this observation, *Propionibacterium* was reported to preferentially colonize the sites rich in sebum-derived lipids such as oleic acid, palmitic acid and mono-glycerides^36^, the density of *P. acnes* populations being positively correlated with the total amount of lipids on skin^37^. Since this species is an important commensal organism of the skin, which benefits the host through secretion of antimicrobial substances, immunomodulation and short chain fatty acid^38^, a depletion of *Propionibacterium* may reduce these skin benefits and thus contribute to clinical/physiological signs of an aged skin.

The LefSE analysis identified the 38 species largely contributing to differentiate the skin microbiomes of the two age groups, most of which were significantly overrepresented in the older samples (Fig. 4 and Supplementary Table S4), and included many normal bacteria frequently detected in the oral cavity^27^. This finding suggests that oral bacteria largely contribute to bacterial diversification and alteration in the older skins. Since the oral species detected was sufficiently high in the relative abundance with high location specificity in the older group, we may exclude the possibility that many of them transiently colonized. Although our data also suggest age-dependent interconnection between skin and oral bacteria, at present, the biological role of these oral bacteria colonizing the skin was largely unknown. On the other hand, it is intriguing that the prevalence of oral bacteria was identified as a signature of microbiome in aged skins in this study as well as that in skins of patients with atopic dermatitis of which the mean age was 23.1^39^.

Overall, our data suggest that the micro-biotope of younger skins exert a stronger selection pressure on the colonization of the skin bacteria than older skins, resulting in a lower inter-individual variability and species richness in younger skin microbiome than the older one. These alterations could be explained by less nutrients available on older skins than in younger skins due to the decrease in skin cells renewal and sweat and sebaceous functions, and by weakened immune system in the older skin^40^.

The correlation analysis between the skin parameters and microbiome data revealed that there are two different patterns of correlation, i.e. age-associated and non age-associated. The age-associated patterns, which showed correlations with both age and some microbial data, were observed with several skin parameters such as sebum level, pore density and skin elasticity-related Ur and Ur/Ue^28^ that decrease with increasing age, and dark spot density and echogenicity of deep dermis that increase with increasing age^41, 42^. These age-associated correlations could illustrate an indirect association through different aging-related phenomena. In contrast, the dark spot area represents the non age-associated parameter that showed no strong association with age but was positively correlated with the Shannon index and negatively with the abundance of *Propionibacterium* and *Prevotella* in our data. The dark spot area is known to increase with sun-exposure^43^, suggesting that it is associated with photoaging of the skin rather than chronological aging. Since photoaging occurs through sun exposure as an environmental factor, further studies comparing the sun-exposed and unexposed skin microbiomes will be of interest to elucidate differences between environmental and host’s intrinsic effects on skin microbiomes.

In summary, this study presented a strong effect of aging on diversification and alterations in the skin microbiome of Japanese women. Our data further found that proliferation of oral normal bacteria largely contributed to the higher microbial diversity in the older than the younger skin microbiome. It can also be suggested that aging-related diversification of skin microbiomes is correlated with changes in skin physiology that depend or not upon aging. Thus, skin microbiomes could be used as a new indicator to quantify skin age irrespective of their chronological age. As a significant correlation between taxonomic and functional diversities has been reported^44^, elucidating functional features specific to older skins by metagenomics and metabolomics will be valuable to more deeply understand aged skin for development of new approaches to prevent skin disorders that specifically occur in older skins^45^.

## Methods

### Subjects and sample collection

The skin samples were collected from 37 healthy Japanese women of two age groups, i.e. 18 subjects of 21–37 years, average 27.3 years and 19 subjects over 60 years (60 to 76, average 67.4 years) living in the suburbs of Tokyo, Japan (Supplementary Table S1).

Informed written consent was obtained from all participants: they were informed the purpose of the study, and written consent was obtained according to the principles of the Declaration of Helsinki. This study was approved by the ethical committees in Nihon L’Oreal and the University of Tokyo.

All of the participants provided information regarding health status, medical history, and daily habits. The volunteers were non-smokers and did not receive antibiotics or systemic antifungals one month prior to sampling. They did not have apparent cutaneous disorders such as acne and atopic dermatitis, nor self-declaration of any of cutaneous manifestation. The volunteers were asked to wash their hair and scalp with the provided shampoo without anti-bacterial compounds for 2 days prior to sampling and not to shampoo or apply any hair care or hair-styling product before the sampling, to standardize the scalp condition. They were asked to remove their makeup and double-washed their faces right after check in, using Lancome’s BI-FACIL and Shu Uemura’s FRESH CLEANSING OIL. BI-FACIL contains benzalkonium chloride and FRESH CLEANSING OIL phenoxyethanol as preservatives. Sampling was conducted in a controlled room at 22 °C, humidity 60%, two hours after the last face wash without applying any skincare product prior to the sampling. During two hours prior to the sampling, volunteers did not talk with each other avoiding contamination of face with saliva through speaking.

The skin area used for sampling was 1 × 2 cm^2^ for scalp, forehead and cheek, and 4 × 10 cm^2^ for forearm. The samples were collected by using sterile cotton-tipped swabs (COPAN Ref.165KS01) pre-moistened with 0.15 M sodium chloride (NaCl), 0.1% Tween 20. Each cotton tip was placed into an Eppendorf tube containing 500 uL of 1X PBS-20% glycerol, immediately frozen in liquid nitrogen, and stored at −80 °C prior to DNA extraction.

### DNA extraction, PCR amplification and sequencing

DNA was extracted from the heads of cotton swabs using the PowerSoil DNA Isolation Kit (MO BIO Laboratories, Inc., California, USA), according to the manufacturer’s protocol. Hypervariable regions (V1-V2) of 16S rRNA gene were PCR-amplified, and multiplexed amplicon pyrosequencing was carried out using 454 GS FLX Titanium or 454 GS JUNIOR (Roche Applied Science) as previously described^46^.

The reads obtained were assigned to samples on the basis of their barcode sequence. Reads with an average quality value <25 and inexact matches to both universal primer were filtered off. For chimera checking and taxonomy assignment of the 16S reads, we used three publically available databases: Ribosomal Database Project (RDP) v. 10.27, CORE (http://microbiome.osu.edu/), and a reference genome sequence database obtained from the NCBI FTP site (ftp://ftp.ncbi.nih.gov/genbank/, December 2011). Reads having BLAST match lengths <90% with the representative sequence in the three databases were considered as chimeras and removed. The reads assigned to eukaryotes in the database (described above) were also removed. Finally, filter-passed reads were used for further analysis after trimming off both primer sequences.

The 16S rRNA gene V1-V2 region sequences analyzed in this study were deposited in DDBJ/GenBank/EMBL with the accession numbers SAMD00057049-SAMD00057196.

### Operational taxonomic unit clustering and taxonomic assignment

From the filter-passed reads, 2,500 high-quality reads/sample were randomly chosen. The total reads were then sorted according to the average quality value^47^, and grouped into operational taxonomic units (OTUs) using UCLUST (http://www.drive5.com/) with a sequence identity threshold of 96%. The representative sequences of the generated OTUs were subjected to homology search against the databases mentioned above using the GLSEARCH program for taxonomic assignments. For assignment at the phylum, genus and species levels, sequence similarity thresholds of 70%, 94% and 96% were applied, respectively. The ratio of the OTUs accurately assigned to particular taxa in the databases was 100% at the phylum level, 61.3% at the genus level, and 46.5% at the species level, respectively.

### Statistical analysis

Statistical significance was determined using one-way Anova for multiple comparisons, the Mann-Whitney *t*-test, the Friedman test, and the Chi-Square test, as appropriate; differences *P* < 0.05 were noted as significant. All analysis was performed using Graph Pad Prism software.

Permutational Multivariate Analysis of Variance (PERMANOVA)^48^ was applied to verify differences based on subject, age (younger vs older) and body site (cheek vs forehead vs scalp vs forearm) using the function “adonis” Vegan package (v 2.2-1) in R software program. PERMANOVA global R^2^ value ranges from 1 to −1 (R ~ 0 indicates the same level of variation within and between groups). Permutation test for homogeneity of multivariate dispersion were performed using the function “betadisper” Vegan package.

Sample average similarity was calculated using SIMPER56. Diversity was measured using the Shannon-Weaver diversity index. These analyses were performed using Primer 657^49^. Rarefaction curves were obtained for each sample using PAST3.0358^50^. Linear discriminant analysis (LDA) effect size tool- LEfSe^25^ was used to identify species with differential relative abundance between the younger and older groups.

### Skin parameter collection

Sebum accumulation on forehead skin was measured by a Sebumeter (SM815, Courage & Khazaka, Germany) 30 min after the face cleansing by following manufacturer’s instruction. Measurement of dark spot area, including any kind of pigmentation disorders such as freckles, melasma and lentigo, was carried out based on the images acquired by Chromasphere video camera (L’Oreal, Chevilly-Larue, France)^51^. Pore area was calculated as a mean value of sizes of outer pore openings observed in the 398 images acquired by DermaScore (Innofaith, Eindhoven, Netherlands) as described in Flament *et al*.^52^. For skin elasticity related parameters, Ur (Immediate retraction of the skin) and Ue (immediate distension of the skin)^28^ were determined using DTM310 Torque Meter (Dia-Stron, Andover, UK) following manufacturer’s instruction. The ratio Ur/Ue was used to represent skin elasticity as described previously^28^. Cheek images were acquired with an ultrasonic Echograph B-scan equipped with a high-frequency broadband transducer working at 25 MHz and providing axial resolution of 75 µm at −6 dB^53, 54^. Skin thickness was measured and the ultrasound characterization of the dermis was quantified with in-house software. The dermis characterization is obtained by extracting two separate rectangular regions of interest (ROI) of equal thickness, each of them equal to 33% of the thickness of the whole skin: ROI “superficial dermis” corresponds to the first third of the whole skin from the surface echo of the skin and ROI “deep dermis” corresponds to the last third of the whole skin from the dermal-hypodermal junction. In these ROI, the average levels of grey were quantified and referred as Echogenicity.

### Data availability

The datasets generated during and/or analyzed during the current study are included in this published article and its Supplementary Information files except the 16S rRNA gene V1-V2 region sequences analyzed in this study which have been deposited in DDBJ/GenBank/EMBL with the accession numbers SAMD00057049-SAMD00057196.

## Electronic supplementary material

Supplementary Information
