# Supplementary material for: Aging-related changes in the diversity of women’s skin microbiomes associated with oral bacteria
Source: Sci Rep. 2017 Sep 5;7:10567. doi: 10.1038/s41598-017-10834-9 (PMC5585242; doi:10.1038/s41598-017-10834-9)
Supplement: Supplementary file 1 — Supplementary Information [file 41598_2017_10834_MOESM1_ESM.pdf]

Title

Aging-related changes in the diversity of women's skin microbiomes associated with oral bacteria

Authors

Nakako Shibagaki[a,1], Wataru Suda[b,c,1], Cecile Clavaud[d], Philippe Bastien[d], Lena Takayasu[b], Erica Iioka[b], Rina Kurokawa[b], Naoko Yamashita[b], Yasue Hattori[b], Chie Shindo[b], Lionel Breton[d,*], Masahira Hattori[b,e,*]

Author affiliation

[a]*Nihon L'Oreal Research & Innovation, KSP, Sakado, Takatsu, Kawasaki, Kanagawa 213-0012, Japan.*

[b]*Laboratory of Metagenomics, Graduate School of Frontier Sciences, The University of Tokyo, 5-1-5 Kashiwanoha, Kashiwa, Chiba 277-8561 Japan.*

[c]*Department of Microbiology and Immunology, Keio University School of Medicine, 35 Shinanomachi, Shinjuku-ku, Tokyo 160-8582, Japan.*

[d]*L'Oreal Research and Innovation, Aulnay-sous-Bois, France.*

[e]*Graduate School of Advanced Science and Engineering, Waseda University, 3-4-1 Okubo Shinjuku-ku, Tokyo 169-8555 Japan*

Corresponding author

Masahira Hattori

Graduate School of Advanced Science and Engineering, Waseda University, 3-4-1 Okubo Shinjuku-ku, Tokyo 169-8555 Japan.

Telephone number: 81-3-5286-3382; E-mail: m-hattori@aoni.waseda.jp

Footnotes

[1]Equally contributing authors

[*]To whom correspondence should be addressed.
Prof. Masahira Hattori, Graduate School of Advanced Science and Engineering, Waseda University, 3-4-1 Okubo Shinjuku-ku, Tokyo 169-8555, Japan.
Tel.: +81-4-7136-4070, Fax: +81-4-7136-4084
E-mail: m-hattori@aoni.waseda.jp
Dr. Lionel Breton, L'Oreal Research and Innovation, Aulnay-sous-Bois, France.
Tel.: +33- 148689627
E-mail LBRETON@rd.loreal.com

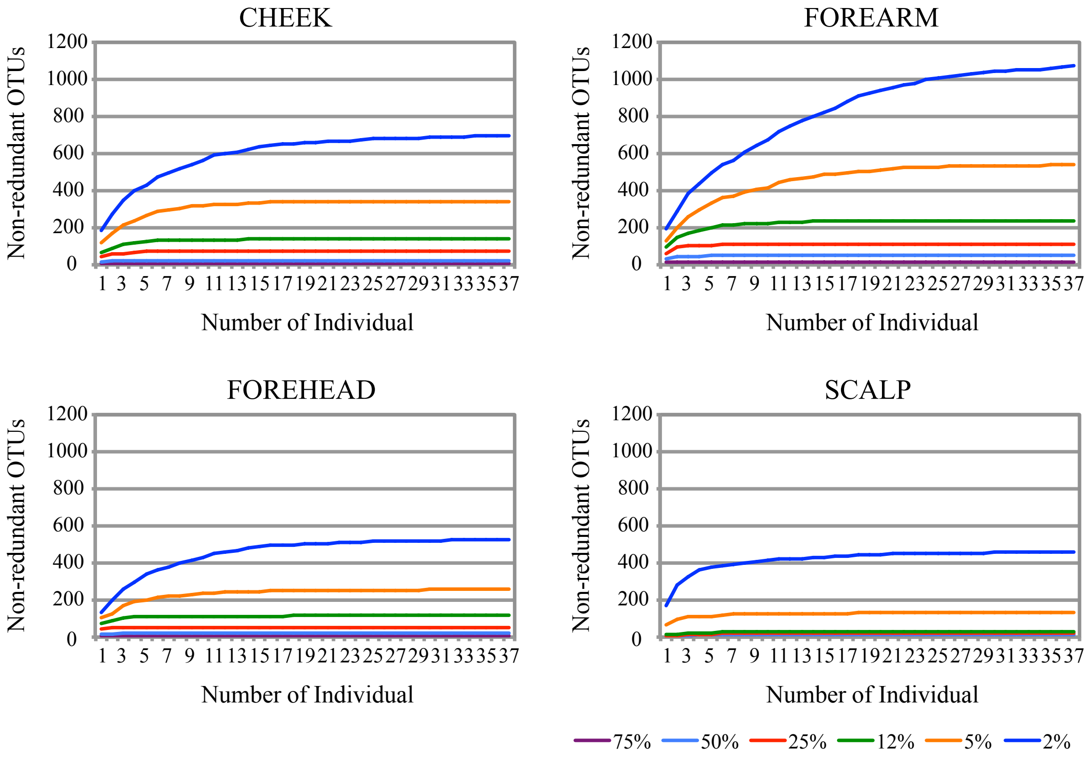

Supplementary Fig. S1. Rarefaction curves of observed OTU numbers in the four skin microbiomes. Samples are colored by the proportion of shared subjects in the population as follows; ≥2% (blue), ≥5% (orange), ≥12% (green), ≥25% (red), ≥50% (light blue), ≥75% (purple). OTUs having ≥0.1% relative abundance were analyzed.

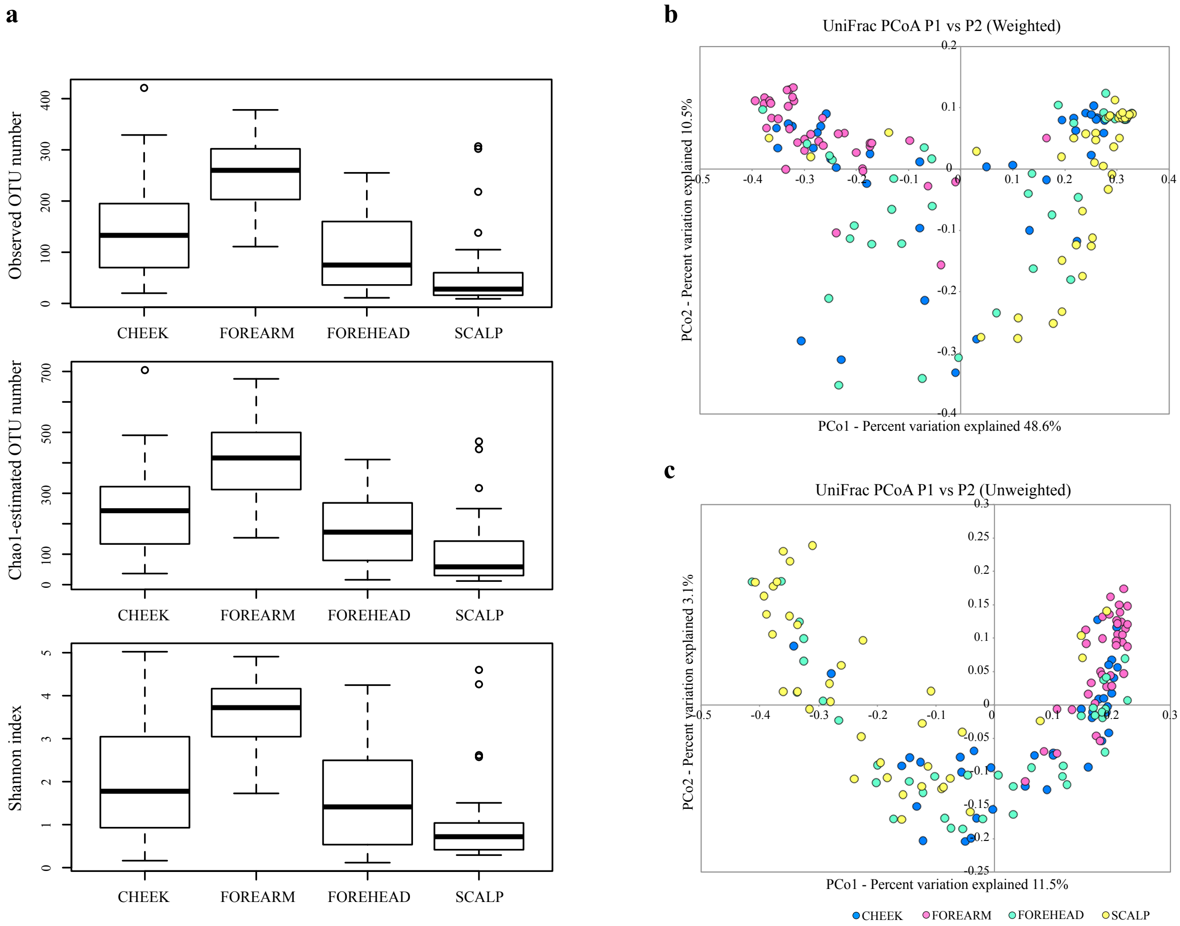

Supplementary Fig. S2. (a) Alpha diversity of the four skin microbiomes based on the observed and Chao1-estimated OTU numbers and the Shannon index. The values are obtained from clustering of 2,500 reads per sample (n=37).

(b,c) Diversity in 148 skin microbiome samples from four different skin sites. The PCoA plots based on the weighted UniFrac (b) and the unweighted UniFrac (c) analysis are shown. Statistical assessment by PERMANOVA and beta dispersion analysis for similarity between the skin microbiomes is summarized in Supplementary Table S3.

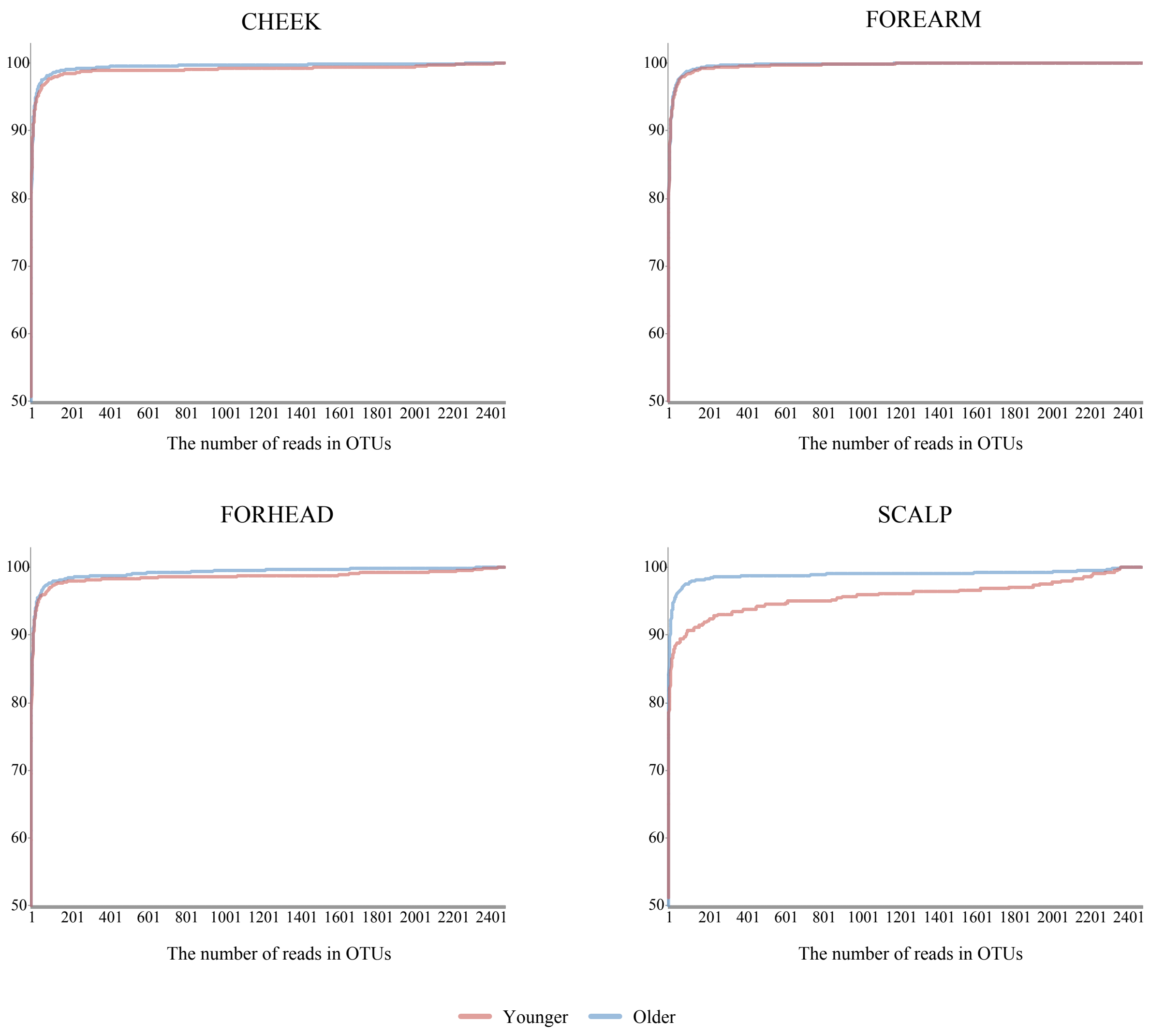

Supplementary Fig. S3. Rarefaction curves of the observed OTU number in each skin site. Proportion of the cumulative OTU numbers with increase in the number of reads in OTUs is shown.

a

b

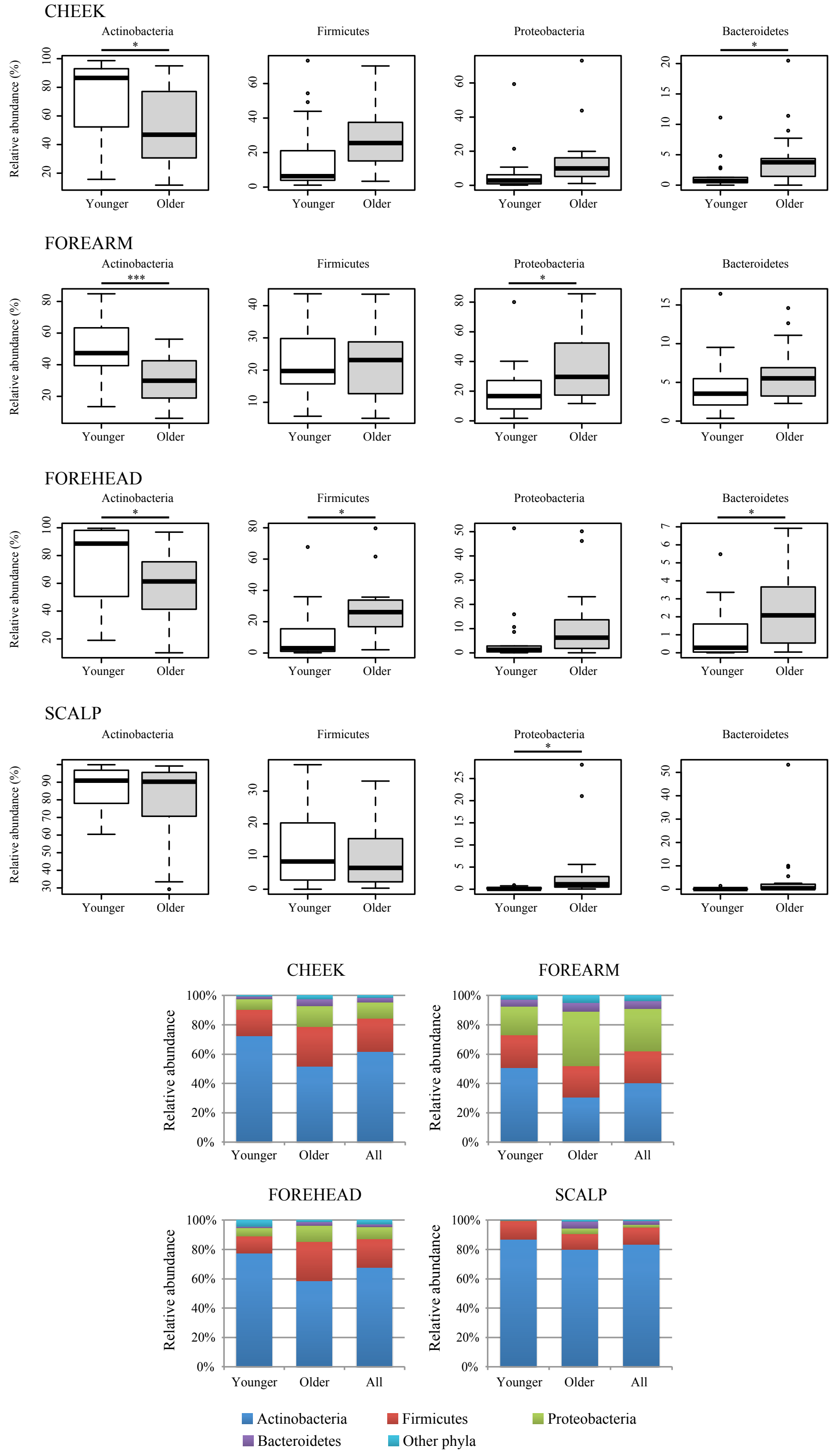

Supplementary Fig. S4. The relative abundance of the major phyla in the four skin microbiomes of all subjects (a), and the older and younger (b). P-values are calculated by Welch's *t* test, and indicate *p <0.05, **p <0.01, ***p <0.001.

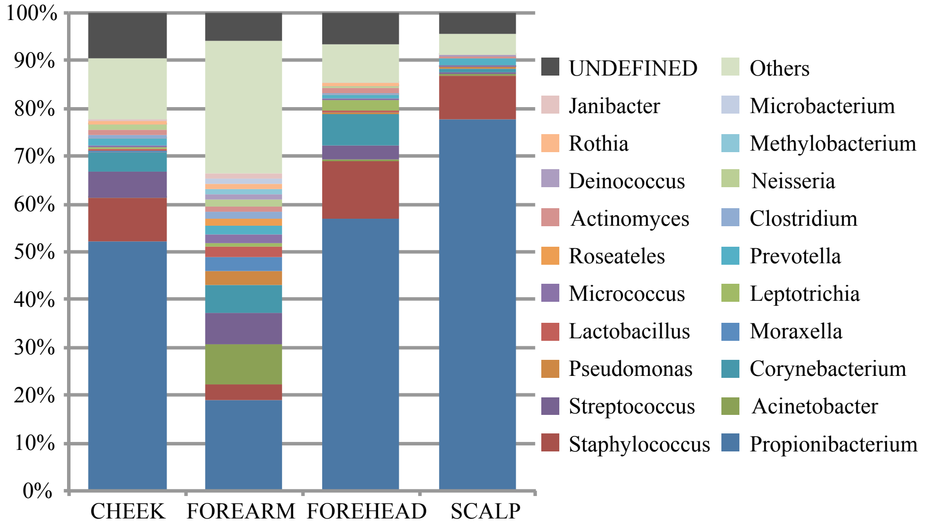

Supplementary Fig. S5. The average relative abundance at the genus level in the four skin microbiomes. The y-axis indicates the relative abundance of each genus. Top 20 dominant genera detected in the all subjects are shown in the right of the figure.

Supplementary Table 1. Subjects and skin clinical metadata analyzed in this

SampleID	Age	Skin site	Cheek												Forehead	
		Method	Chromasphere		Echography			Dermascore			Dermal Torque Meter			pH meter	Sebumeter	pH meter
		Age group	Dark Spot Area (cm^2)	Dark Spot Density (spot number/cm^2)	Thickness_skin (mm)	Echogenicity at superficial dermins	Echogenicity at deep dermis	Pore density (pore number/cm^2)	Pore area, average (mm^2)	Largest Pore Area (mm2)	Ue	Ur	Ur/Ue	pH	Sebum level 30min after face wash	pH
1	60	Older	1.27	4.89	1.72	11.42	11.5	39.265897	0.54475	5.293663	5.42	1.18	0.22	5.9	98	5.5
2	61	Older	2.1	2.34	1.91	18.36	8.1	32.746849	0.438911	2.910098	5	1.04	0.21	5.2	2	4.3
3	76	Older	7.19	0.73	1.75	16.63	12.87	38.053051	0.36753	2.424033	2.94	0.92	0.31	5.8	1	4.8
4	28	Younger	0.59	1.78	1.96	18.9	9.72	41.539984	0.558197	4.167304	4.1	1.54	0.38	5.7	35	6
5	22	Younger	2.65	0.21	1.53	15.67	9.05	26.379406	0.324356	1.787786	2.66	0.86	0.32	5.3	15	4.6
6	68	Older	1.97	1.33	1.53	20.04	15.29	35.93057	0.360841	1.384455	3.08	0.88	0.29	5.7	50	5.1
7	60	Older	3.16	0.67	1.55	18.37	8.64	41.539984	0.447488	2.404248	5.3	1.12	0.21	5.7	0	4.7
8	69	Older	1.3	6.49	1.5	9.71	13.1	44.5721	0.490634	3.760376	4.68	1.04	0.22	5.7	0	5
9	31	Younger	2.48	0.33	2.06	16.38	8.54	44.875311	0.553284	4.335921	5.26	1.58	0.3	6	70	5
10	74	Older	2.17	5.03	1.97	16.47	11.37	.	.	.	5.54	1.32	0.24	4.8	30	4.8
11	21	Younger	2.34	1.67	1.78	22.02	7.74	44.420494	0.382983	3.161449	4.86	1.74	0.36	6	24	5.2
12	21	Younger	1.46	2.22	1.58	25.99	8.89	44.420494	0.342807	1.683918	3.26	1.1	0.34	6	18	5.1
13	37	Younger	0.98	1				46.391369	0.413914	6.33414	2.8	1.06	0.38	5.7	37	5.4
14	73	Older	1.73	6.28	1.73	20.45	14.91	42.601225	0.40347	1.893003	5.2	1	0.19	5.6	2	5.2
15	66	Older	1.98	2.22	1.87	16.02	8.97	35.93057	0.582348	3.598953	3.82	0.98	0.26	5.8	3	4.8
16	74	Older	1.3	4.82	1.92	13.38	10.16	34.414512	0.756732	11.217728	3.24	0.82	0.25	5.5	3	4.6
17	71	Older	1.71	3	1.8	16.94	13.6	37.446628	0.531704	4.860206	3.4	1.28	0.38	5.5	34	5.1
18	65	Older	2.74	0.22	1.74	9.87	6.26	37.598234	0.532468	3.322422	4.54	0.92	0.2	5.5	13	5.2
19	36	Younger	0.81	2				36.385388	0.654907	4.048598	4.56	1.16	0.25	4.8	68	4.4
20	21	Younger	1.92	0.22				42.904436	0.48179	4.118742	4.69	1.61	0.34	5.1	39	4.6
21	23	Younger	1.21	1.11	1.76	22.65	5.54	43.814071	0.341778	1.018444	4.56	1.56	0.34	5.4	3	4.7
22	21	Younger	1.37	2.34				45.026917	0.533946	3.93169	4.48	1.76	0.39	5.3	31	5.1
23	70	Older	1.02	4.29	1.99	17.14	9.91	35.627359	0.697326	6.343583	3.72	0.96	0.26	5.5	39	4.7
24	69	Older	2.02	1.89	1.71	21.51	7.91	40.478744	0.425219	2.688424	4.78	0.88	0.18	5.2	1	4.8
25	25	Younger	1.58	0.44	1.64	15.32	6.89	45.63334	0.375223	1.586795	4.28	1.22	0.29	5.8	29	5.4
26	60	Older	1.2	3.45	1.55	14.74	7.96	39.872321	0.486811	3.026107	5.48	1.16	0.21	5.3	37	4.7
27	66	Older	1.2	3.14	1.63	17.79	14.18	41.236773	0.503397	2.245074	3.68	1.18	0.32	5.4	1	4.7
28	76	Older	1.73	3.98	1.81	16.94	14.04	34.86933	0.433666	2.218096	4.44	1.12	0.25	5.6	2	5
29	35	Younger	2.71	0.78	1.74	12.98	5.82	46.391369	0.424169	1.968543	4.08	1.24	0.3	5.5	14	5.5
30	35	Younger	2.47	0.89	1.72	16.61	7.87	39.265897	0.437039	2.214498	5.56	1.46	0.26	5.7	15	5.2
31	21	Younger	1.29	2.56				50.484725	0.336462	1.519798	5.04	1.16	0.23	5.6	16	4.9
32	35	Younger	3.27	0.33	1.67	17.15	8.83	38.962686	0.343376	1.115118	5.06	1.58	0.31	5.8	46	5.5
33	28	Younger	2.64	0.44	1.62	9.07	5.16	49.878302	0.456922	3.759476	6.14	1.9	0.31	5.2	31	5
34	22	Younger	1.01	1.11	1.94	20.28	7.15	46.088158	0.463663	2.522055	4.32	1.46	0.34	5.6	25	4.9
35	63	Older	2.38	1.68	1.72	17.92	13.52	41.539984	0.495167	3.107492	2.68	1.04	0.39	4.9	6	4.9
36	60	Older	2.14	4.08	1.84	23.22	9.77	36.385388	0.747863	6.066602	3.02	0.92	0.3	5.4	3	5.3
37	29	Younger	1.25	2.45				44.420494	0.443429	1.860628	3.84	1.26	0.33	5.8	37	5.3
		Average of Older	2.12	3.19	1.75	16.68	11.16	38.34	0.51	3.82	4.21	1.04	0.26	5.47	17.11	4.91
		Average of Younger	1.78	1.22	1.75	17.75	7.60	43.18	0.44	2.84	4.42	1.40	0.32	5.57	30.72	5.10
		p value (t test)	0.35124	0.00030	0.99285	0.50041	0.00007	0.00315	0.03775	0.13989	0.49877	0.00005	0.00096	0.34862	0.06741	0.09522

Supplementary Table 2. The number of OTUs generated from clustering of 16S reads

Subject	Cheek	Forearm	Forehead	Scalp	All skin sites
Older	1,611	1,919	1,193	1,192	3,441
Younger	1,024	2,090	753	313	2,300
All subjects	1,955	2,940	1,424	1,285	4,156

Supplementary Table 3. Evaluation of UniFrac distances between the four skin sites by PERMANOVA and beta dispersion analysis

Category		Permanova				Betadisper	
		Weigthed		Unweigthed		Weigted	Unweigted
		R^2	P value	R^2	P value	P value	P value
Overall	n=148	0.0847	0.000999	0.0390	0.000999	0.001*	0.001*
CK vs FAR	CK (n=37)<br>FAR (n=37)	0.1381	0.000999	0.0413	0.000999	0.872	0.001*
CK vs FH	CK (n=37)<br>FH (n=37)	0.0119	0.4765	0.0200	0.03596	0.806	0.103
CK vs SC	CK (n=37)<br>SC (n=37)	0.1231	0.000999	0.0636	0.000999	0.002*	0.001*
FAR vs FH	FAR (n=37)<br>FH (n=37)	0.1948	0.000999	0.0657	0.000999	0.865	0.001*
FAR vs SC	FAR (n=37)<br>SC (n=37)	0.3414	0.000999	0.1239	0.000999	0.001*	0.001*
FH vs SC	FH (n=37)<br>SC (n=37)	0.0531	0.000999	0.0424	0.000999	0.001*	0.016*

CK: Cheek; FH: Forehead; FA: Forearm; SC: Scalp

*Statistical significance: P ≤0.05 by the beta dispersion analysis

Supplementary Table 4. The 38 species/OTUs having a significant change in the abundance between the younger and older groups

Genus	Species/OTUs	**LDA Score (log10)				***Average abundance (%)	***Average frequency (%)	****CORE ID#
		Cheek	Forearm	Forehead	Scalp			
Acinetobacter	*Acinetobacter baumannii*				3.21	0.824	23.6	6
Arthrobacter	*Arthrobacter davidanieli*	3.53		3.92		0.414	54.7	0
Corynebacterium	*Corynebacterium pseudogenitalium*	3.81		4.35		0.772	58.8	0
	Corynebacterium tuberculostearicum			3.86		0.475	53.4	1
	Corynebacterium segmentosum	3.71		3.54		0.103	27.0	1
Delftia	*Delftia* sp. Cs1-4			4.02	4.0	0.649	55.4	1
Finegoldia	*Finegoldia magna*			3.87		0.427	48.6	0
Fusobacterium	*Fusobacterium nucleatum*	3.83				0.119	32.4	3
Gemella	*Gemella haemolysans*	3.72				0.317	38.5	2
Granulicatella	*Granulicatella adiacens*		4.01	3.64		0.209	35.1	4
Haemophilus	*Haemophilus parainfluenzae*				3.21	0.102	32.4	7
Klebsiella	*Klebsiella pneumoniae*	4.29	3.93	3.67	3.28	0.164	29.1	5
Kocuria	*Kocuria palustris*		3.85			0.119	16.9	0
Moraxella	*Moraxella osloensis*			3.29	3.17	0.782	46.6	6
Neisseria	*Neisseria mucosa*		4.01			0.321	41.9	2
	Neisseria macacae	3.99		3.18	3.89	0.264	41.2	2
Prevotella	*Prevotella* sp. CD3_34	3.84	3.91	3.63	3.45	0.266	41.9	7
Propionibacterium	*Propionibacterium acnes*	4.97	5.15	5.15		47.697	100.0	1
	OTU03449 *Propionibacterium acnes* (95.9%)*			3.45		0.486	79.7	0
	Propionibacterium sp. 434-HC2	3.7	4.15			0.256	62.2	1
	Propionibacterium granulosum	3.79		3.24		0.391	58.8	1
	Propionibacterium sp. MSP09A		3.96			0.088	32.4	0
Pseudomonas	*Pseudomonas rhizosphaerae*		4.35			0.378	17.6	1
Rhodococcus	*bromate-reducing bacterium* B7		4.14			0.228	26.4	2
Rothia	*Rothia mucilaginosa*	3.67	3.86		3.71	0.264	41.9	5
	Rothia aeria	3.86	3.80		3.64	0.196	39.9	3
Staphylococcus	*Staphylococcus epidermidis*			4.60		7.551	93.9	1
	Staphylococcus warneri			3.34	3.40	0.162	44.6	1
	Staphylococcus hominis			3.39	3.72	0.184	31.8	2
Streptococcus	*Streptococcus salivarius/thermophilus*	4.15	4.37	3.87		0.858	58.1	4
	Streptococcus parasanguinis	3.95	4.17	3.68		0.217	41.2	4
	Streptococcus sanguinis	3.86				0.208	39.2	6
	Streptococcus sp. I-P16				3.30	0.143	33.1	4
	Streptococcus genomosp. C8		3.93	3.48		0.148	27.7	4
	Streptococcus gordonii	4.25	4.08			0.090	25.0	4
UNDEFINED	OTU00099 *Corynebacterium confusum* (93.2%)*		3.87		4.24	1.099	45.3	0
	OTU00339 *Clostridium symbiosum* (90.7%)*	4.32		4.08		0.482	35.1	0
Veillonella	*Veillonella parvula*	4.02	4.05	3.95		0.301	41.2	11

*Values in parentheses indicate 16S rRNA gene sequence identity with that of the given species.

**The species increased in the older and younger group are marked by grey and magenta, respectively.

***Values indicate average abundance of the species and proportion of subjects having the species, respectively.

****Number of CORE IDs hit with ≥97% identity and ≥90% coverage.
